# A Needle in a Haystack: Looking for Gaps in Treatment and Education in Emergency Medicine—Editorial

**DOI:** 10.3390/healthcare13202598

**Published:** 2025-10-15

**Authors:** Filip Jaskiewicz, Klaudiusz Nadolny

**Affiliations:** 1 Department of Emergency Medical Services, Medical University of Lodz, 90-419 Lodz, Poland; 2 Department of Emergency Medical Service, Faculty of Medicine, Silesian Academy in Katowice, 40-555 Katowice, Poland; prrm.knadolny@interia.pl; 3 Collegium Medicum, WSB University, 41-300 Dabrowa Gornicza, Poland

## 1. Introduction

When this Special Issue, “A Needle in a Haystack: Looking for Gaps in Treatment and Education in Emergency Medicine,” was first announced, its aim was to highlight the unique nature of emergency medicine as a discipline. Emergency medicine addresses life-threatening conditions across all age groups and aetiologies, often under severe time constraints and with limited resources [1]. It spans the entire continuum of care—from first aid provided by bystanders to advanced, multi-speciality interventions in emergency departments—requiring not only clinical excellence but also well-coordinated logistics, education, and system-level solutions to ensure optimal patient outcomes [2].

As noted in the original call for papers, conducting research in this field presents significant methodological and organisational challenges. Despite technological advances and growing scientific interest, many clinical recommendations still rely on weak evidence or expert consensus rather than robust data [3,4]. Moreover, the diversity and complexity of emergency medicine leave important questions underexplored, contributing to persistent gaps between guidelines and real-world practice [5].

Emergency medicine is also strongly shaped by global challenges such as ageing populations, climate-related disasters, armed conflicts, and pandemics, all of which increase demands on prehospital and hospital systems [6,7]. Addressing these issues requires high-quality, context-sensitive research that can be translated into practice.

The purpose of this Special Issue was to encourage researchers to think “outside the box,” address knowledge gaps through innovative study designs and generate evidence with clear implications for patient care. Now, after ten articles have been published, this editorial summarises their key contributions, extracts cross-cutting insights, and outlines priorities for future research, education, and system development in emergency medicine [8].

## 2. Overview of Contributions

(1) ““Even Though the System Had Failed Him His Entire Life, We Were Failing Him Yet Again”: How Clinical, Welfare, and Penal Medicine Interact to Drive Health Inequities and Medical Moral Injury.”

A case study from Los Angeles examines how penal medicine, welfare systems, and clinical care intersect to perpetuate health inequities for justice-involved patients with injection-drug-use-related infective endocarditis. Physician narratives highlight moral injury when institutional constraints prevent equitable care. The authors call for restorative justice programmes, expanded harm-reduction services (including in carceral settings), and explicit institutional protocols for managing structurally vulnerable patients (Contribution 1).

(2) “Assessment of the Severity of COVID-19 Based on Examination and Laboratory Diagnostics in Relation to CT Imagery—Single-Centre Study.”

This observational study links admission findings to subsequent CT-assessed lung involvement in COVID-19 patients. Significant associations were found between initial BNP, HCO_3_^−^, and base excess levels and severity of lung involvement, suggesting that simple laboratory and clinical markers can guide early risk stratification before imaging results are available (Contribution 2).

(3) “Successful Intraosseous Adenosine for Termination of SVT in a 3.5-Year-Old—Case Report and Literature Review.”

A case report is presented of a 3.5-year-old child with supraventricular tachycardia (SVT) who was successfully treated using intraosseous (IO) adenosine after failed vagal manoeuvres. Sinus rhythm was restored after the second dose without recurrence. The case underscores IO administration as a viable alternative when intravenous access is difficult in paediatric emergencies (Contribution 3).

(4) “CALL TO ECLS—Acronym for Reporting ECPR Candidates from Prehospital Setting to Destination Centres.”

The CALL TO ECLS acronym was validated as a prehospital communication tool for patients considered for extracorporeal CPR. Expert surveys showed high clarity, utility, and importance (I-CVI 0.87–0.97; S-CVI-AVE > 0.9), though the limited response rate was acknowledged as a study limitation. The revised version added explicit guidance on recognising “signs of life” (e.g., ROSC, motor response) during CPR to improve decisional clarity (Contribution 4).

(5) “What Mistakes Can Be Made When Performing Electrical Cardioversion?—Analysis of Emergency Medical Team Performance during Championships”

An analysis of competition scenarios (2015 and 2019) demonstrated high accuracy in initial qualification for electrical cardioversion in unstable tachycardia but declining adherence with successive shocks—especially for the re-activating synchronisation mode and selecting appropriate energies—while safety measures remained consistent. The findings highlight the need for refresher training and cognitive aids under time pressure (Contribution 5).

(6) “The Role of Paramedics in Diagnosing Sandifer’s Syndrome—Case Report”

A 7-week-old with seizure-like episodes was ultimately diagnosed with Sandifer’s syndrome, a rare manifestation of GERD often misdiagnosed as epilepsy. This case illustrates paramedics’ role in early recognition through careful history-taking and assessment, preventing unnecessary anti-epileptic treatments (Contribution 6).

(7) “Uterotonic Drugs in Prevention and Management of Postpartum Haemorrhage in Prehospital Deliveries—Systematic Review.”

A systematic review identified only four eligible studies on prehospital use of uterotonic agents, despite postpartum haemorrhage being a leading cause of maternal mortality. The evidence gap highlights the need for standardised EMS protocols and further research on uterotonic availability, dosing, and outcomes in prehospital settings (Contribution 7).

(8) “Adult Triage in the Emergency Department: Introducing a Multi-Layer Triage System.”

Greek emergency departments implemented a multi-layer triage system integrating elements of established triage frameworks and early-warning scores (ESI, NEWS, HEART). This system aims to reduce under-triage, improve prioritisation, and offer flexibility while maintaining structured, reproducible decision-making (Contribution 8).

(9) “Medical Students’ Knowledge and Adherence to Paediatric Choking Rescue Manoeuvre Guidelines—Multicentre Study.”

A multicentre survey across 12 universities in Canada, Libya, and Poland (290 analysed responses) revealed substantial variability in knowledge retention regarding paediatric choking management, including body positioning, blind finger sweeps, and post-event follow-up. The findings argue for standardised, evidence-based curricula in paediatric first aid (Contribution 9).

(10) “Management of Polytraumatised Patients: Challenges and Insights into Air Transfer.”

Analysis of 77 polytrauma patients transported by air in Romania (mean age 17.3 years; 74% road traffic accidents; 2:1 male predominance) calls for prevention strategies tailored to patient profiles and better risk analysis protocols to guide decisions on air versus ground transport efficiency (Contribution 10).

## 3. Cross-Cutting Themes

- Skills and systems integration: Technical proficiency (e.g., cardioversion, IO drug delivery) must be reinforced by reliable communication tools (e.g., CALL TO ECLS) and decision frameworks (e.g., multi-layer triage).
- Standardisation vs. local adaptation: Evidence gaps in uterotonic access and choking management contrast with validated tools like CALL TO ECLS, illustrating where global standards versus local tailoring are appropriate.
- Equity and moral injury: Justice-involved care and maternal health gaps reveal how emergency systems can perpetuate inequities unless welfare support, harm-reduction, and clear clinical protocols are integrated.
- Prehospital criticality: From air transfer to obstetric haemorrhage, early decisions in EMS repeatedly shape downstream outcomes, supporting targeted investment in prehospital education and infrastructure.

## 4. Future Priorities

1. Curriculum harmonisation for paediatric emergencies with longitudinal skill-retention studies.
2. Tool implementation research linking CALL TO ECLS use to ECMO activation times and survival.
3. High-reliability training for cardioversion, airway management, and CPR choreography.
4. Prehospital obstetric care trials testing uterotonic access, dosing, and outcomes.
5. Transport modality research comparing air vs. ground transfer outcomes using functional metrics.
6. Equity-focused interventions addressing harm reduction, re-entry support, and moral injury metrics.

## 5. Conclusions

This Special Issue collectively demonstrates that addressing gaps in emergency medicine requires a systems-oriented approach, bridging education, clinical practice, policy, and research. By integrating validated communication tools, harmonising training curricula, and prioritising equity-focused interventions, healthcare systems can become more resilient and responsive to emerging threats. Future efforts should foster international collaboration through multicentre trials, global clinical registries, and cross-border training initiatives to ensure that evidence-based practices reach diverse healthcare settings. Such coordinated actions can accelerate progress toward reducing preventable deaths, strengthening preparedness for mass-casualty events, and embedding continuous quality improvement across the entire chain of survival.
